# Immune responses of honeybees and their fitness costs as compared to bumblebees

**DOI:** 10.1007/s13592-014-0318-x

**Published:** 2014-10-17

**Authors:** Ulrike Riessberger-Gallé, Javier Hernández López, Wolfgang Schuehly, Sara Crockett, Sophie Krainer, Karl Crailsheim

**Affiliations:** Department of Zoology, Universitätsplatz 2, Karl-Franzens University of Graz, A-8010 Graz, Austria

**Keywords:** *Apis*, American foulbrood, *Bombus*, flight performance, immune challenge

## Abstract

Immune responses of invertebrates imply more than developing a merely unspecific response to an infection. Great interest has been raised to unveil whether this investment into immunity also involves fitness costs associated to the individual or the group. Focusing on the immune responses of honeybees, we use the well-studied insect bumblebee for comparison. Bumblebees are capable of producing specific immune responses to infections whereas this has not been assessed for honeybees so far. We investigated whether a prior bacterial encounter provides protection against a later exposure to the same or a different bacterium in honeybees. Additionally, we studied whether the foraging activities of honeybees and bumblebees are affected upon immune stimulation by assessing the flight performance. Finally, the acceptance behavior of nestmates toward immune-challenged honeybees was determined. Results show that despite stimulating the immune system of honeybees, no protective effects to infections were found. Further, honeybees were not affected by an immune challenge in their flight performance whereas bumblebees showed significant flight impairment. Immune-challenged honeybees showed lower survival rates than naive individuals when introduced into a regular colony. Here, we reveal different immune response-cost scenarios in honeybees and bumblebees for the first time.

**Electronic supplementary material:**

The online version of this article (doi:10.1007/s13592-014-0318-x) contains supplementary material, which is available to authorized users.

## Introduction

During recent years, an increasing number of studies have highlighted the variety of immune responses and their associated fitness costs in invertebrates (Rowley and Powell 2007; Moret and Schmid-Hempel 2000; Zanchi et al. 2011; Roth and Kurtz 2009a, b; Rodrigues et al. 2010; Korner and Schmid-Hempel 2004). While some have reported that immune responses in invertebrates are not as highly conserved as previously believed, others are reporting fitness costs associated to these responses (Alghamdi et al. 2008; Mallon et al. 2003; Sadd and Schmid-Hempel 2009). For example, experiments with bumblebees (*Bombus terrestris*) demonstrated that their immune system is capable of responding to a pathogenic infection in a specific way, when a previous encounter with the same type of pathogen had previously occurred (homologous exposure) (Sadd and Schmid-Hempel 2006). In addition to the specificity of the response, it was observed to persist over a period of 3 weeks in bumblebees, which also suggests the development of a kind of immunological memory. This was the first report that invertebrates are capable of producing immune responses as complex as those observed in vertebrates—albeit by different means. Other investigations carried out with the red flour beetle (*Tribolium castaneum*) reported and corroborated similar results as those in bumblebees (Roth and Kurtz 2009a, b). An immune stimulus prior to a pathogen encounter using the same bacterial strain (homologous exposure) led to a higher survival rate of *T. castaneum* than it was the case when two different bacterial strains were used for the immune stimulus and the later pathogen encounter (heterologous exposure).

Fitness costs comprise the energetic investment that single individuals make into their own immune defense and costs for social changes among nestmates in the colony (Alghamdi et al. 2008; Mallon et al. 2003; Sadd and Schmid-Hempel 2009). Considering individual costs, authors hypothesize that they may exhibit malfunctions while performing particular tasks during immune activation due to a diversion of metabolic resources. For example, free-flying bumblebees showed impairment in learning the color of rewarding flowers when their immune system was stimulated non-pathogenically (Alghamdi et al. 2008). The non-pathogenic stimulation of the immune system allowed an exclusion of any adverse effect stemming from the parasite, which would have inevitably had a direct effect on the host’s behavior. Hence, the authors linked the observed learning impairment to the effects from the immune activation. Other experiments investigating associative learning in honeybees have drawn similar conclusions. Honeybees injected with lipopolysaccharides (LPS), which are known to trigger a strong immune reaction, showed reduced ability to associate an odor with a sugar reward (Mallon et al. 2003). The authors concluded that the immune response interferes with learning skills and memory formation.

In keeping with this concept, further research in honeybees has revealed that infections of forager bees with deformed wing virus (DWV) cause learning deficits (Iqbal and Mueller 2007). Infected individuals showed increased responsiveness to sucrose and impairments in associative olfactory learning. Other investigations on honeybees demonstrated that foragers parasitized by *Varroa destructor* needed either a longer time to return to the colony, returned at a lower frequency, or did not return at all, indicating impaired orientation abilities (Kralj and Fuchs 2006).

Costs at the colony level due to changes in social interactions among healthy nestmates and infected nestmates include enhanced grooming behavior toward infected individuals, hygienic behavior (e.g., removal or cannibalism of diseased brood), enhanced aggression behavior toward infected workers to exclude them from the nest, or necrophoric behavior to remove dead workers (Evans and Spivak 2010). As an example, the injection of ringer or LPS in honeybee workers induced significant changes in the chemical profile of cuticular hydrocarbons that affect social interactions between unhealthy and healthy nestmates (Richard et al. 2008; Richard et al. 2012).

Here, we aim to reveal to which extent the performance of the immune system of honeybees can be compared to that of bumblebees from three different perspectives:

1. Enhanced protection upon second exposure to pathogens
  We determined whether the first exposure to a pathogen produces a benefit upon a second encounter to the same pathogen or a different pathogen. To confirm activation of the immune system of injected bees, the antimicrobial activity of the hemolymph was measured using a zone of inhibition assay.
2. Fitness costs (if existing) related to an investment into immunity
  Here, we compared costs associated to immune activation between honeybees and bumblebees by investigating their flight performance (Brodschneider et al. 2009).
3. Survival of immune-stimulated honeybees introduced in regular colonies

Sets of naive, ringer-injected, and LPS-injected honeybees were introduced into observation colonies, and their survival was followed over 25 days. This allowed us to determine the degree of acceptance of treated individuals by healthy nestmates.

We demonstrate that honeybees and bumblebees display important differences in their immune responses when an infection is present and, consequently, differences in fitness costs related to these responses.

## Material and methods

### Experiments carried out at the individual level

All honeybees (*Apis mellifera carnica*) used for experiments were taken from healthy queen right full colonies located at the University of Graz. Commercially available bumblebee colonies of *Bombus terrestris* (Natupol, Koppert) were maintained at the University of Graz and used for flight performance experiments.

#### Challenge and infection experiments with honeybees

For this purpose, we first exposed honeybee callow workers to different species of heat-killed bacteria (challenge) and injected the same bees 1 week later with viable bacteria (infection) in all possible combinations (i.e., homologous and heterologous).

##### Challenge

Experiments were carried out using freshly emerged honeybees randomly mixed from at least three colonies. During the challenge, honeybees were chilled on ice for 5–10 min and injected using a Hamilton microliter syringe between the fifth and sixth abdominal tergites. Bees designated as immune-challenged received either 2 μL of heat-killed (90 °C, 10 min) vegetative forms of the Gram(+) bacterium *Paenibacillus larvae* (*Pl*) genotype Eric II (strain 233/00), the Gram(−) bacterium *Escherichia coli* (*Ec*) (ATCC 25922) or the Gram(+) bacterium *Staphylococcus aureus* (*Sa*) (ATCC 29213) suspended in sterile ringer solution at a concentration of 10^8^ bacterial cells mL^−1^. This dose causes a strong immune stimulation in the individual, which has been described previously for bumblebees (Sadd and Schmid-Hempel 2007) or *Drosophila* (Pham et al. 2007).

To confirm the lack of growth of heat-killed bacteria, samples were plated out on MYPGD agar (15 g yeast extract, 10 g Müller-Hinton-Bouillon, 2 g glucose, 3 g K_2_HPO_4_, 1 g pyruvic acid sodium salt, 15 g Agar Nr. 1, 1,000 mL distilled water, pH 7.4). Bacterial species were selected in order to have one natural honeybee pathogen (*Pl*) and bacteria of a different Gram type. As control, a group of bees that received 2 μL of sterile ringer solution and a group composed of naive bees were also included. All bee groups were kept in separate cages in an incubator (34.5 °C, 60 % humidity) and supplied with water, sucrose solution, and pollen ad libitum (Alaux et al. 2010).

This design allowed assessing the immune response upon a second encounter with a pathogen. In addition, we could study the ability of honeybees to develop a specific immune response with regard to different bacteria species.

The mortality rate was recorded once at the end of the week before the infection experiment started and used to assess any effect of the challenge in the survival of the individuals among groups.

##### Infection

At day 8 after the challenge, bees were injected for a second time with either 2 μL of ringer saline solution, 2 μL of viable *Pl* bacteria (2 × 10^5^ cells/mL), *Ec* (1 × 10^6^ cells/mL), or *Sa* (1 × 10^4^ cells/mL). Bacterial doses had been previously selected and were adjusted to kill infected naive bees gradually within 1 to 5 days, rather than killing within hours (see supplementary material, Table S1).

In a fully reciprocal design, all challenge treatments were combined with all infection treatments. Final experimental groups were as follows: ringer-challenged bees were injected for a second time with ringer as a control (R-R, *n* = 83), viable *Pl* (R-*Pl*, *n* = 129), *Ec* (R-*Ec*, *n* = 51), or *Sa* (R-*Sa*, *n* = 47). *Pl*-challenged bees were injected for a second time with ringer (*Pl*-R, *n* = 71), viable *Pl* (*Pl*-*Pl*, *n* = 129), viable *Ec* (*Pl*-*Ec*, *n* = 26), or viable *Sa* (*Pl*-*Sa*, *n* = 47). *Ec*-challenged bees were injected for a second time with ringer (*Ec*-R, *n* = 41), viable *Pl* (*Ec*-*Pl*, *n* = 28), viable *Ec* (*Ec*-*Ec*, *n* = 31), or viable *Sa* (*Ec*-*Sa*, *n* = 38). *Sa*-challenged bees were injected for a second time with ringer (*Sa*-R, *n* = 32), viable *Pl* (*Sa*-*Pl*, *n* = 27), viable *Ec* (*Sa*-*Ec*, *n* = 34), or viable *Sa* (*Sa*-*Sa*, *n* = 38). Survival data were now recorded during the subsequent 5 days for all groups. For the most relevant combinations (R-*Pl* and *Pl*-*Pl*, also R-R and R-*Pl*), four replicates (i.e., times the experiment was repeated) were conducted, and for the rest of groups, two replicates were accomplished using honeybees from at least three colonies. Replicates per group were tested for differences and later combined for final statistical analysis (see supplementary material, Table S2).

During statistical analysis, we first investigated whether the challenge type showed an effect on the survival of the four initial bee groups (ringer-, *Pl*-, *Ec*-, or *Sa*-challenged). For this purpose, we recorded the mortality of bees at the end of the week after the challenge and until the infection experiment was carried out. Additional analyses were run to determine whether honeybees showed a difference in survival when exposed to the same bacterial species for a second time (homologous) or when exposed to a different bacterial species (heterologous).

#### Monitoring of bacterial proliferation in the hemolymph of honeybees

Here, we aimed to determine whether the death rate of bees infected with *Pl* correlated with the proliferation rate of bacteria in the hemolymph.

Twenty-four hours after the infection process was carried out, hemolymph of bees of the following groups (N-*Pl*, *n* = 17; R-*Pl*, *n* = 14; R-R, *n* = 6; *Pl*-R, *n* = 6; and *Pl*-*Pl*, *n* = 23) was taken and plated on MYPGD agar. Infected bees were chilled on ice for 5–10 min, and 1 μL of hemolymph was withdrawn using 5-μL volume glass microcapillaries and added to 0.5-mL Eppendorf vials containing 200 μL of saline buffer. One hundred microliters of this solution (corresponding to 0.5 μL of hemolymph) was immediately plated on MYPGD-agar, and colony-forming units (CFU) were counted 24 h later.

The mortality of these bees during the infectious phase (described in Sect. 2.1.1) was expected to correlate directly with bacterial proliferation in the hemocoel.

#### Zone of inhibition assay with hemolymph of challenged honeybees

Activation of an immune response in challenged individuals was confirmed by assessing antibacterial activity of the hemolymph. Hemolymph of naive honeybees (*n* = 6), ringer-challenged (*n* = 5), and heat-killed *Pl*-challenged honeybees (*n* = 8) was used in a zone of inhibition assay. For this purpose, we placed 2 μL of extracted hemolymph into 2-mm diameter holes punched into a 1 % nutrient agar (10 g bacto-tryptone, 5 g yeast extract, 10 g NaCl, 1,000 mL distilled water, pH 7–7.5) already containing *Pl* adjusted to 10^5^ cells/mL of medium. The diameter of the clearing zones was measured after 24 h of incubation at 25 °C.

#### Flight performance of honeybees and bumblebees after immune challenge

Honeybees and bumblebees were immune-stimulated by injection of heat-killed *Pl* bacteria, and 24 h later, their flight performance was quantitatively evaluated on a roundabout by measuring overall flight duration, covered distance, maximum speed reached, and flight average speed. Honeybee brood combs of different colonies were incubated at 34.5 °C and 60 % humidity. Emerging bees were marked and introduced into one regular colony located at the University of Graz. For flight experiments, marked bees returning from flights with pollen loads were collected at the hive entrance. All honeybees used in this experiment were 15 to 20 days old. Bumblebees were collected at the entrance of the colonies once they returned from pollen-collecting flights. This way, we assured that foragers were selected for the experiment. Honeybees and bumble bees were randomly assigned to one of the following groups: naive, ringer-injected, or heat-killed *Pl*-injected. Injections were performed as described in Sect. 2.1.1. Subsequently, the three groups of bees were kept in separate cages for 24 h and then used to assess their flight performance.

Flight efficiency tests were carried out as follows: bees were attached to the rotator arm of a roundabout and performed a first flight that served as an “emptying flight.” This depletes the existing energy reserves in the honey sac of the bee. After the emptying flight, bees of all three experimental groups were fed with 10 μL of a 2 M glucose solution. They were then allowed to rest for a period of 5 min and subsequently attached to the rotator arm of the roundabout to start the flight. A 60-W lamp was used for illumination and to regulate ambient temperature in the roundabout to 26–27 °C. All data generated during the flight were automatically recorded. At the end of the flight, data for flight duration, covered distance, maximum speed, and flight average speed were gathered.

### Experiments carried out at the colony level

#### Survival of naive, ringer-injected, and LPS-injected honeybees in colonies

Combs of four different colonies containing capped brood were incubated at 34.5 °C and 60 % humidity. Emerged honeybees were mixed, assigned to one of the following groups: naive, ringer-injected, or LPS-injected (0.5 mg/mL of solution), and marked individually. Since this experiment was carried out in regular colonies with free-flying bees, the use of *Pl* bacteria was avoided and replaced by LPS, which is known to strongly stimulate an immune response in honeybees and other insects (Laughton et al. 2011; Haine et al. 2008). Two microliters of ringer or LPS were injected per bee (described in Sect. 2.1.1. Following injections, bees were placed back into the incubator for 2 h to recover from chilling on ice and to discard any dead bees. Bees that were damaged (e.g., gut tissue punctured and damaged during injection) perish normally within minutes to an hour. Once we discarded dead bodies (~1 every 150), all remaining honeybees were introduced in two observation colonies (colony 1—naive = 348, ringer-injected = 265, LPS-injected = 277; colony2—naive = 343, ringer-injected = 342, LPS-injected = 331). These colonies are composed of three combs situated in a vertical disposition and two glass panels that allow observation of all individuals. During the next 25 days, daily screenings of marked bees were carried out in order to obtain survival data for the three experimental groups. Every single individual was individually marked. Screenings were done twice a day, beginning in the morning (8:30 a.m.) and in the afternoon (4 p.m.) during one and a half hour every time and for both colonies at the same time. Screenings were done by two persons at each hive and repeated as many times as possible during the observation period. Individuals were considered alive if they were seen at least one time a day or overlooked but observed the following day.

### Statistical analysis

The software package SPSS v. 19 was used for statistical analyses. For challenge and infection experiments, a Pearson chi-squared test was conducted to assess any effect of the challenge in mortality among groups before the infection. A Cox regression analysis was later performed to estimate differences in mortality after the infection of individuals. For flight performance experiments, an analysis of variance (ANOVA) was carried out to test for the influence of weight in flight parameters according to the insect (honeybees vs bumblebees) and treatment (naive, ringer, and *Pl*). Furthermore, an analysis of covariance (ANCOVA) was performed for the flight parameters (flight duration, covered distance, maximum speed, and average speed) by insect and treatment, covarying for weight. A test of the homogeneity of the regression slopes was conducted. Because four flight parameters were analyzed, a Bonferroni correction was done for the significances of the ANCOVAs. As a measure of effect size, the partial eta-squared coefficient was computed. Means and standard deviations are reported as observed (unadjusted) values for weight and adjusted for the covariate values for the flight parameters. The level of significance was determined at 5 %. Finally, a Cox regression analysis was conducted to test for the survival of naive, ringer-injected, and LPS-injected honeybees in colonies.

## Results

### At the individual level

#### Challenge and infection experiments with honeybees

The survival rate of honeybees during the week following the challenge treatment did not differ between groups according to the type of challenge (Pearson $\chi$ ^2^ *df* = 3, *p* = 0.788) (see supplementary material, Table S3). Considering the effect of the challenge type in the survival rate of honeybees upon a second encounter with a pathogen, a Cox regression analysis revealed no statistically significant differences between groups, regardless of the type of challenge or infection ($\chi$ ^2^ *df* = 9, *p* = 0.144) (Table I).

**Table I:** A Cox regression analysis showed no effects of first bacterial exposure (challenge) on survival of honeybees upon reinfections (infection).

	Wald’s $\chi$ ^2^	*df*	*p* value	Relative risk	95 % CI for relative risk	
					Lower	Upper
Challenge	0.869	3	0.833			
Infection	105.614	3	0.000			
*Pl* infection	55.818	1	0.000	83.894	26.246	268.164
*Ec* infection	75.211	1	0.000	184.495	56.738	599.923
*Sa* infection	78.689	1	0.000	216.045	65.877	708.530
Challenge × infection	13.420	9	0.144			

These results indicate that the immune system of honeybee workers seems to be incapable of reacting in a stronger manner to second pathogen encounters, i.e., the survival rate of individuals is not enhanced in either homologous or heterologous exposure experiments.

#### Monitoring of bacterial proliferation in the hemolymph of honeybees

Hemolymph samples of *Pl*-infected bees plated on agar revealed bacterial proliferation in all individuals of all groups. A Kruskal-Wallis test revealed significant differences in CFUs obtained from hemolymph of N-*Pl*, R-*Pl*, or *Pl*-*Pl* bees (*p* = 0.008). CFUs in the hemolymph of N-*Pl* honeybees showed non-significant differences from R-*Pl* bees (*p* = 0.922). CFUs in the hemolymph of R-*Pl* bees did not differed from *Pl*-*Pl* bees (*p* = 0.057). Nevertheless, N-*Pl* bees showed significant differences in CFUs from *Pl*-*Pl* bees (*p* = 0.030). Even though a slower proliferation of bacteria in *Pl*-*Pl* individuals was observed in comparison to R-*Pl*, which may contribute to the non-significantly longer life expectancy observed (see supplementary material, Table S4), the presence of bacterial proliferation in the hemolymph of all individuals examined here confirms that mortality was caused by the infection.

#### Size of the zone of inhibition using hemolymph of challenged honeybees

Activation of the immune system of honeybees was confirmed by means of a zone of inhibition test. A Kruskal-Wallis test revealed significant differences in the size of clearing zones obtained from hemolymph of naive, ringer-injected, or heat-killed *Pl*-injected bees (*p* < 0.001). Hemolymph of naive honeybees produced a mean inhibition zone of 4.2 mm as compared to 9.4 mm of *Pl*-stimulated bees (Bonferroni post hoc comparisons, *p* < 0.001). Inhibition zones of *Pl*-stimulated bees also differed from ringer-injected bees (Bonferroni post hoc comparisons, *p* < 0.001, mean inhibition zone of ringer-injected bees 6.8 mm) and between naive and ringer-injected honeybees (Bonferroni post hoc comparisons, *p* < 0.001) (see supplementary material, Table S5)

We did not carry out inhibition zone assays for individuals challenged with heat-killed *Ec* or *Sa* or the verification of mortality of individuals infected with viable *Ec* or *Sa*, but this should not affect the significance of our results. Challenge and infections of individuals were performed in the exactly same manner for all bacterial species.

#### Flight performance of honeybees and bumblebees after immune challenge

An ANOVA test was conducted using weight as dependent variable to test for its influence in treatment and the interaction of insects by treatment. Statistically significant differences in weight with a strong effect between honeybees and bumblebees were found (*F*(1, 67) = 188.92, *p* < .001, partial eta^2^ = 0.74). The average weight of bumblebees (*M* = 0.23 g, SD = 0.05) was higher than that of honeybees (*M* = 0.12 g, SD = 0.01; see supplementary material, Table S6).

**Figure 1:**
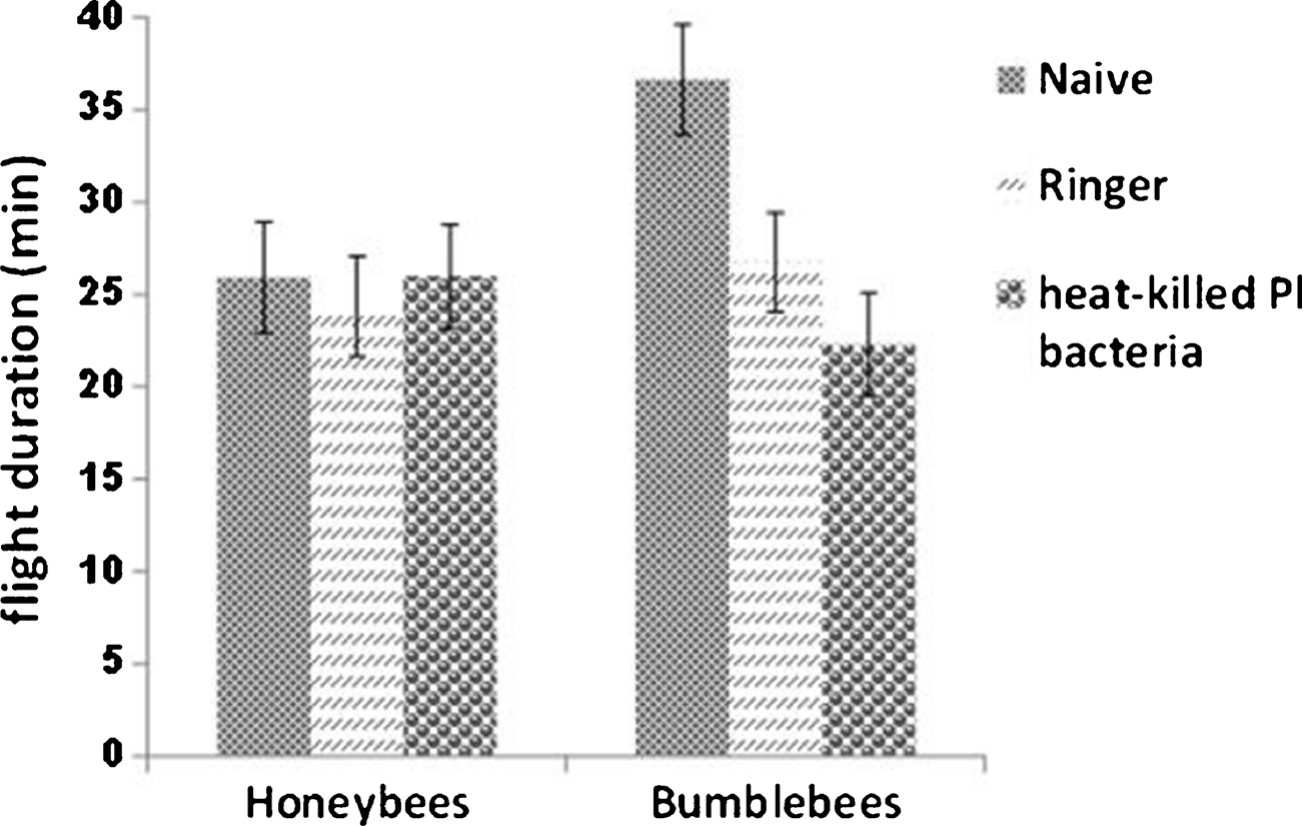
Flight duration in minutes of honeybees (naive = 15, ringer = 13, heat-killed *Pl* bacteria = 14) and bumblebees (naive = 10, ringer = 11, heat-killed *Pl* bacteria = 12). Significant differences were found between the naive and the *Pl* bumblebee groups.

**Figure 2:**
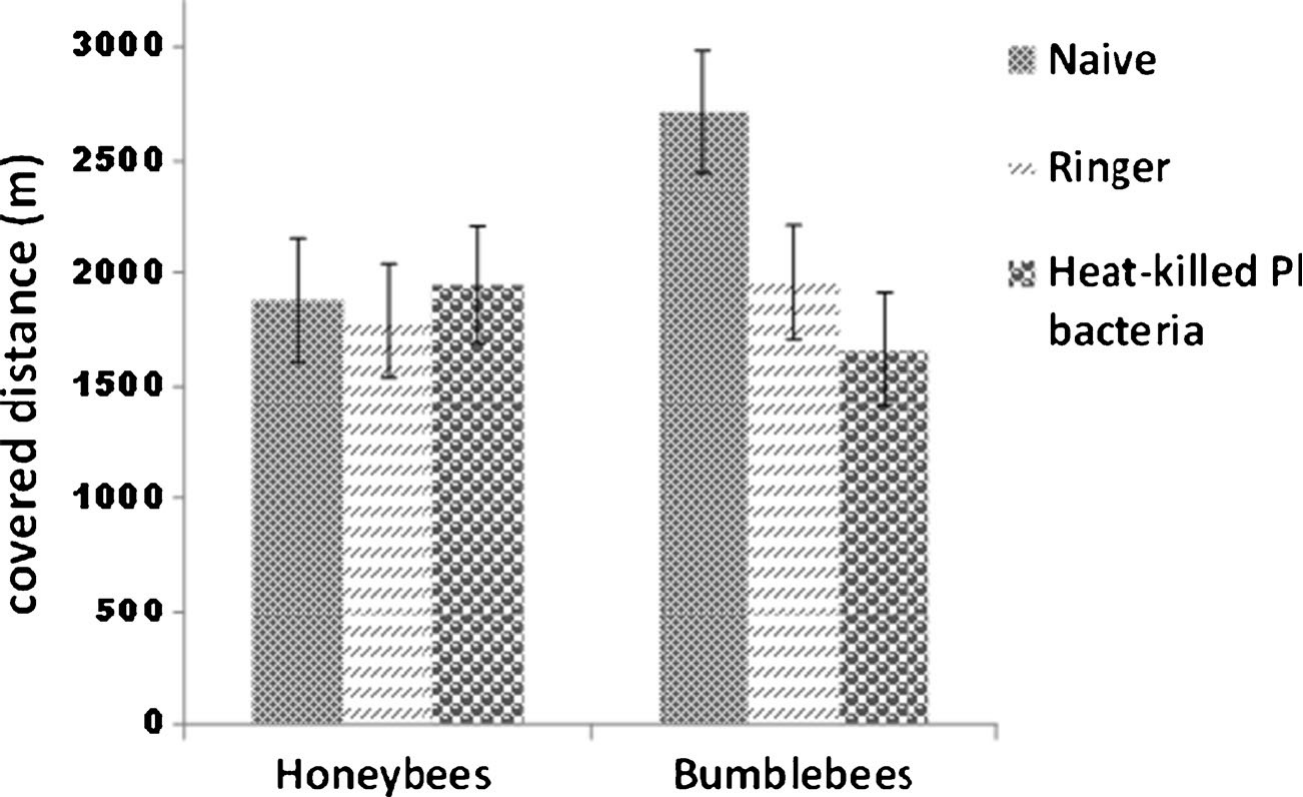
Covered distance in meters of honeybees (naive = 15, ringer = 13, heat-killed *Pl* bacteria = 14) and bumblebees (naive = 10, ringer = 11, heat-killed *Pl* bacteria = 12).

Nevertheless, no effect of weight was found for treatment (*F*(2, 67) = 1.18, *p* = .31, partial eta^2^ = 0.03) and the interaction of insect by treatment (*F*(2, 67) = 2.54, *p* = .09, partial eta^2^ = 0.07). Therefore, weight was used as a covariate in the subsequent ANCOVA tests. Detailed results from the ANCOVA tests for the flight parameters by insect and treatment, covarying for weight, are shown in Table II (adjusted means with SDs and 95 % confidence levels) (see also Figures 1 and 2 and supplementary material, Table S7). From all parameters tested (flight duration, covered distance, maximum speed, and averaged speed), flight duration was statistically significantly affected by insect treatment (*F*(2, 66) = 4.89, *p* = 0.010) with a medium-sized effect (partial eta^2^ = 0.13). Flight duration is affected by the treatment regarding insect species. Bonferroni-corrected post hoc comparisons revealed that within bumblebees, the flight duration was significantly longer in the naive group (adjusted mean = 36.62 min) than in the ringer-treated group (adjusted mean = 26.74) or *Pl*-treated group (adjusted mean = 22.28) (Figure 1). Pairwise comparisons by treatment revealed that, whereas wounding of individuals (ringer group) is not enough to produce a significant difference to the naive group (*p* = 0.066, SD = 2.44), wounding and injection of heat-killed bacteria (*Pl* group) produces a significant difference to the naive group (*p* = .009, SD = 2.32). No significant differences were observed between the ringer and the *Pl* group (*p* = 1.000, SD = 2.37). No significant differences were found for honeybees in any of the flight parameters. Interestingly, with the same amount of sugar solution, the flight duration of bumblebees was longer than in honeybees (adjusted mean = 36.62 min and 25.91 min, respectively, *P* = .033, SD = 5.05), probably due to the higher amount of reserves in the larger insect. Also of interest, a statistically significant effect of weight on covered distance (*F*(1, 66) = 8.50, *p* = 0.005) with a medium-sized effect (partial eta^2^ = 0.11) was detected. Within each insect species, individuals with a higher weight covered shorter distances than lighter individuals. Homogeneity of the regression slopes was given (F(5, 61) = 0.68, *p* = 0.64).

**Table II:** Results of analysis of covariance for flight parameters by insects (honeybee vs bumblebee) and treatment (naive, ringer, and *Pl*) covarying for weight.

Source of variation	*F*	*df*	*p* value	Partial eta^2^
Dependent variable: flight duration				
Weight	6.30	1, 66	0.015	0.09
Insect	0.70	1, 66	0.406	0.01
Treatment	5.17	2, 66	0.008	0.14
Insect by treatment	4.89	2, 66	0.010	0.13
Dependent variable: covered distance				
Weight	8.50	1, 66	0.005	0.11
Insect	0.51	1, 66	0.479	0.01
Treatment	3.17	2, 66	0.048	0.09
Insect by treatment	3.72	2, 66	0.030	0.10
Dependent variable: maximum speed				
Weight	0.02	1, 66	0.883	0.00
Insect	0.81	1, 66	0.372	0.01
Treatment	1.20	2, 66	0.307	0.04
Insect by treatment	0.45	2, 66	0.640	0.01
Dependent variable: averaged speed				
Weight	2.58	1, 66	0.113	0.04
Insect	0.00	1, 66	0.990	0.00
Treatment	0.84	2, 66	0.435	0.03
Insect by treatment	0.34	2, 66	0.712	0.01

### At the colony level

#### Survival of naive, ringer-injected, and LPS-injected honeybees in colonies

**Figure 3:**
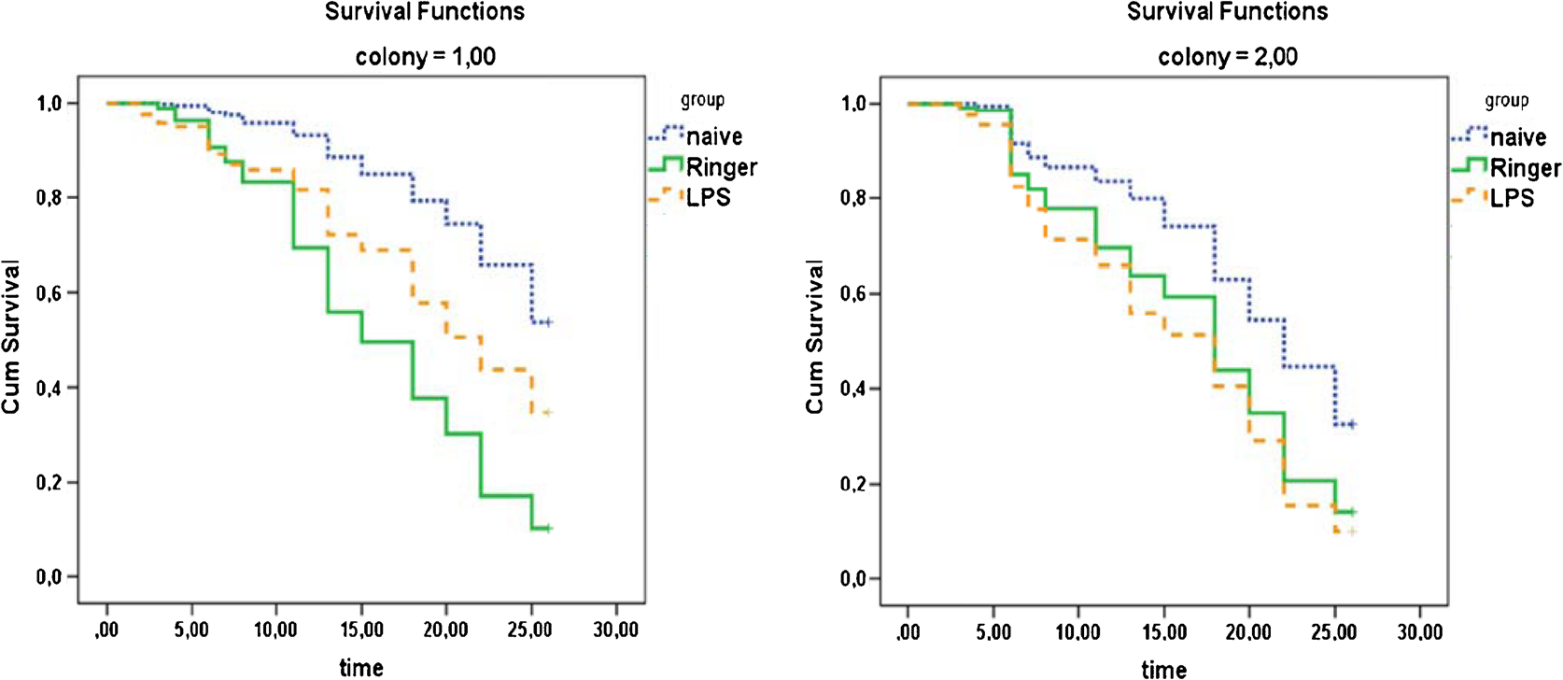
Cumulative survival of naive, ringer-injected, and LPS-injected bees introduced in observational colonies. Colony 1—naive = 348, ringer-injected = 265, LPS-injected = 277. Colony2—naive = 343, ringer-injected = 342, LPS-injected = 331.

Here, we focused on investigating the modulation of social interactions observed between nestmates and immune-challenged individuals. One-day-old marked naive, ringer-injected, and LPS-injected bees from four different colonies were introduced into two observation colonies (1 and 2), and their survival rate was monitored over the next 25 days (Figure 3). To rule out any effect of injection on mortality, in a prior experiment, we kept naive, ringer-injected, and LPS-injected callow workers in plastic cages in an incubator and found no differences between groups in the survival over the time observed (data not shown).

Regarding survival data obtained over the 25-day observation period in colonies 1 and 2, a Cox regression analysis revealed statistically significant differences between naive bees and ringer- or LPS-injected bees (treatment; $\chi$ ^2^ = 85.443, *df* = 2, *p* = 0.000). Further interpretation of the data also showed that both ringer and LPS injections cause a reduction in the survival profile of these bees as compared to naive bees ($\chi$ ^2^ = 74.875, *p* = 0.000, and $\chi$ ^2^ = 4.615, *p* = 0.032, respectively). Nevertheless, our analysis also demonstrated an effect of colony on the survival profile of ringer-injected bees, pointing to different survival profiles of ringer-injected bees depending on the host colony ($\chi$ ^2^ = 27.039, *p* = 0.000). For LPS-injected bees, these effects were not observed ($\chi$ ^2^ = 0.433, *p* = 0.51) (Table III).

**Table III:** A Cox regression analysis revealed effects of the treatment type on the survival profile of ringer- and LPS-injected honeybees in observation colonies.

	Wald’s $\chi$ ^2^	df	*p* value	Relative risk	95.0 % CI for relative risk	
					Lower	Upper
Colony	31.559	1	0.000	1.780	1.456	2.177
Treatment	85.443	2	0.000			
ringer	74.875	1	0.000	6.877	4.444	10.644
LPS	4.615	1	0.032	1.652	1.045	2.612
Colony × treatment	45.532	2	0.000			
Colony × ringer	27.039	1	0.000	0.496	0.381	0.646
Colony × LPS	0.433	1	0.510	1.096	0.834	1.440

Colony 1—naive bees, *n* = 348; ringer-injected, *n* = 265; LPS-injected, *n* = 277. Colony 2—naive bees, *n* = 343; ringer-injected, *n* = 342; LPS-injected, *n* = 331

## Discussion

In our research, we first investigated whether honeybees, as a consequence of a previous immunological insult, showed an immune priming that provided protection upon secondary exposure to the same pathogen or a different one. This effect of immune priming has been reported from bumblebees where lower mortality rates of individuals are found after they encounter the same pathogen for a second time (Sadd and Schmid-Hempel 2006). Authors concluded that bumblebees develop specificity in their immune response, which is also accompanied by the development of an immunological memory. The mechanisms underlying these responses are still not fully understood, but results from the literature indicate that hemocytes play a critical role (Roth and Kurtz 2009a, b; Rodrigues et al. 2010).

Honeybee literature shows an upregulation of genes encoding for several antimicrobial peptides during immune activation upon an infectious process (Evans 2004; Cornman et al. 2013; Laughton et al. 2011). This may explain the slightly longer life expectancy (n.s.) observed for *Pl*-*Pl* individuals than for R-*Pl* individuals (4 and 3 days, respectively) (see supplementary material, Table S4) that we observed in our challenge and infection experiments, which is also corroborated by the significant difference in CFUs in the hemolymph of *Pl*-*Pl* individuals compared to N-*Pl* individuals.

These results suggest that there may be an immune stimulation 1 week after the challenge. However, the non-significant differences for *challenge* × *infection* obtained from the analysis of our data (see Table I) are suggesting a much lower immune capability of an individual honeybee’s response in comparison to bumblebees and an immune stimulation not strong enough to affect individual worker survival. Hence, bumblebees and honeybees have apparently evolved immune systems that use different strategies to cope with infections.

Our results suggest that honeybees do not possess immune mechanisms like those of bumblebees and have developed other strategies during the course of evolution. In recent years, different studies have documented that honeybees possess only one third of the genes encoding for immune defense effector proteins in their genome as compared to *Anopheles* or *Drosophila* (Evans et al. 2006). This has been attributed to the development of social immunity in honeybees, which reduces the cost of maintaining pathogen resistance at the individual level (Cremer et al. 2007). Honeybees also collect actively antimicrobial compounds from their environment that can increase immune defense at colony level, e.g., propolis constituents (Simone et al. 2009). Thus, social immunity provides a collective mechanism that keeps the risk of infection to a minimum in an environment with very close physical contact (i.e., honeybee colonies). In this situation, we presume that with the evolution of social immunity, complex immune reactions at the individual level (immune priming and immunological memory) have been reduced to a minimum in honeybees and substituted by a new and more efficient strategy. Whenever social immunity reduces the risks of infections effectively, more complex and likely more costly responses are no longer needed (Wilson-Rich et al. 2009). Whether this is a common trend in highly sociable insects or whether it refers to the particular case of honeybees remains to be disclosed by future investigations on different taxa of insects (primitive vs highly eusocial species) together with whole genome analyses and gene expression studies during immune activation.

Our results from flight performance tests suggest that the energy requirements of the honeybee immune system during activation are not deleterious to their flight capabilities. Bumblebees, however, show impairment in their flight capabilities upon immune activation. Several authors reported learning deficits and loss of orientation associated to infections in honeybees (Mallon et al. 2003; Iqbal and Mueller 2007; Kralj and Fuchs 2006). Although the observed malfunction of the nervous systems does not necessarily need to be connected with our work on flight parameters, we found no fitness costs associated to immune activation in flight performance in honeybees, unlike in bumblebees. Flight experiments were carried out 24 h post-injection of bees and not at different times post-injection, which could had have an influence in the results. Nevertheless, stimulation of the immune system which results in phenoloxidase activity or the expression of antimicrobial peptides in other species of insects reaches the highest point 12 to 48 h post-injection. Thus, a different result in flight performance at a later time post-injection seems unlikely at least for honeybees (Korner and Schmid-Hempel 2004, Haine et al. 2008).

It has been documented that the proportion of chemicals expressed in the cuticle of honeybees increases during infection and that this causes rejection of infected individuals by other nestmates. It was also found that changes in the cuticle were significant both for ringer- and for LPS-injected bees as compared to naive bees (Richard et al. 2008). This might explain the different survival profile of ringer- and LPS-injected honeybee workers found in our experiments carried out in observation colonies. Our long-term experience conducting behavioral studies allows us to affirm that the grade of acceptance of callow workers in a foreign colony can greatly differ. This might explain the difference in the survival profile observed for the ringer group between the colonies, which showed a lower survival rate of ringer-injected bees versus LPS-injected bees in colony A. Nevertheless, all treated bees showed lower survival rates than naive bees. It became apparent that injected honeybees, independently of the effect of wounding or injection of LPS, showed lower survival rates than naive bees. Besides rejection of treated bees by healthy nestmates, other explanations must be considered to account for the observed decrease of treated bees in both colonies. For instance, immune-stimulated workers (ringer and LPS) may begin foraging sooner than naive workers, which will shorten their life expectancy. Alternatively, treated bees may also have abandoned their social function and remove themselves from the colony or may have succumbed to secondary infections (Richard et al. 2008, Rueppell et al. 2010). Data obtained from our investigations do not allow us to exclude any of the likely explanations.

Bumblebee colonies are composed of a few hundred workers compared to thousands of workers in a honeybee colony; therefore, it is likely that losses of bumblebee workers have a higher significance in the development of the colony than losses of honeybee workers. The evolution of a highly efficient immune system could be evolutionarily favored in bumblebees, despite the high cost to the individual (Wilson-Rich et al. 2009), which could prevent such losses in case of infection. In the case of honeybees, it seems that the best strategy that has evolved is an efficient system of social immunity, whereas only queens preserve more complex and costly immune reactions that can be passed on to offspring, conferring them with enhanced responses (Hernández López et al. 2014).

## Electronic supplementary material

Below is the link to the electronic supplementary material.

ESM 1 (DOCX 42 kb)
