# Supplementary material for: Immune responses of honeybees and their fitness costs as compared to bumblebees
Source: Apidologie. 2014 Oct 17;46(2):238–49. doi: 10.1007/s13592-014-0318-x (PMC4579911; doi:10.1007/s13592-014-0318-x)
Supplement: Supplementary file 1 — (DOCX 42 kb) [file 13592_2014_318_MOESM1_ESM.docx]

**APIDOLOGIE**

Immune responses of honeybees and their fitness costs as compared to bumblebees

Ulrike Riessberger-Gallé, Javier Hernández López*, Wolfgang Schuehly, Sara Crockett, Sophie Krainer, Karl Crailsheim

Department of Zoology, Universitätsplatz 2, Karl-Franzens University of Graz, A-8010 Graz, Austria.

(*)Correspondence to: javier.hernandez-lopez@uni-graz.at

**Supplementary Material**

**Table S1.** Preliminary results showing the killing of honeybees by different bacteria at different doses

| **Naive bees** | **Dose of 2µl injection (cell/ml)** | **Mortality per day** | | | | | **Total deads** | **Alive** |
| --- | --- | --- | --- | --- | --- | --- | --- | --- |
|  |  | **d1** | **d2** | **d3** | **d4** | **d5** |  |  |
|  | ***Ec* 10^6^ (32 bees)** | 26 | 2 | 1 | 0 | 0 | 29 | 3 |
|  | *Ec* 10^5^ (22 bees) | 2 | 1 | 2 | 1 | 0 | 6 | 16 |
|  | *Ec* 10^4^ (22 bees) | 0 | 0 | 0 | 0 | 0 | 0 | 22 |
|  | *Sa* 10^5^ (31 bees) | 25 | 4 | 1 | 0 | 0 | 30 | 1 |
|  | ***Sa* 10^4^ (32 bees)** | 17 | 9 | 2 | 1 | 2 | 31 | 1 |
|  | ***Pl* 10^5^ ( 109 bees)** | 0 | 22 | 82 | 5 | 0 | 109 | 0 |
|  | *Pl* 10^4^ ( 30 bees) | 0 | 1 | 20 | 9 | 0 | 30 | 0 |

**Table S2.** Survival data for all groups divided by replicates and Cox regression analysis for replicate level differences

| **Challenge** | **Infection (r=replicate)** | **Mortality per day** | | | | | **Total deads** | **Alive** | **Cox Regression** | | | | | |
| --- | --- | --- | --- | --- | --- | --- | --- | --- | --- | --- | --- | --- | --- | --- |
|  |  | **d1** | **d2** | **d3** | **d4** | **d5** |  |  |  |  |  |  |  |  |
|  | Dose of 2µl injection (cell/ml) |  |  |  |  |  |  |  | B | SE | Wald | df | *Sig.* | Exp(B) |
| **Ringer** | *Ec*10^6^ (n=29)r1 | 21 | 3 | 2 | 3 | 0 | 29 | 0 | .201 | .284 | .499 | 1 | *.480* | 1.222 |
|  | *Ec*10^6^ (n=25)r2 | 18 | 2 | 0 | 2 | 0 | 22 | 3 |  |  |  |  |  |  |
|  | *Sa*10^4^ (n=25)r1 | 10 | 14 | 1 | 0 | 0 | 25 | 0 | .039 | .292 | .018 | 1 | *.893* | 1.040 |
|  | *Sa*10^4^ (n=22)r2 | 8 | 13 | 1 | 0 | 0 | 22 | 0 |  |  |  |  |  |  |
|  | *Pl*10^5^ (n=35)r1 | 0 | 11 | 23 | 1 | 0 | 35 | 0 |  |  | .161 | 3 | *.984* |  |
|  | *Pl*10^5^ (n=30)r2 | 0 | 9 | 21 | 0 | 0 | 30 | 0 |  |  |  |  |  |  |
|  | *Pl*10^5^ (n=34)r3 | 0 | 8 | 25 | 1 | 0 | 34 | 0 |  |  |  |  |  |  |
|  | *Pl*10^5^ (n=30)r4 | 0 | 8 | 22 | 0 | 0 | 30 | 0 |  |  |  |  |  |  |
|  | | | | | | | | | | | | | | |
| ***Sa*** | *Ec*10^6^ (n=18)r1 | 6 | 5 | 3 | 4 | 0 | 18 | 0 | .143 | .344 | .172 | 1 | *.679* | 1.153 |
|  | *Ec*10^6^ (n=16)r2 | 4 | 5 | 2 | 5 | 0 | 16 | 0 |  |  |  |  |  |  |
|  | *Sa*10^4^ (n=22)r1 | 6 | 11 | 3 | 2 | 0 | 22 | 0 | -.157 | .336 | .218 | 1 | *.640* | .855 |
|  | *Sa*10^4^ (n=16)r2 | 5 | 8 | 3 | 0 | 0 | 16 | 0 |  |  |  |  |  |  |
|  | *Pl*10^5^ (n=16)r1 | 0 | 5 | 9 | 2 | 0 | 16 | 0 | -.077 | .392 | .039 | 1 | *.843* | .925 |
|  | *Pl*10^5^ (n=11)r2 | 0 | 4 | 6 | 1 | 0 | 11 | 0 |  |  |  |  |  |  |
|  | | | | | | | | | | | | | | |
| ***Ec*** | *Ec*10^6^ (n=17)r1 | 9 | 3 | 3 | 2 | 0 | 17 | 0 | .105 | .363 | .084 | 1 | *.771* | 1.111 |
|  | *Ec*10^6^ (n=14)r2 | 7 | 3 | 1 | 3 | 0 | 14 | 0 |  |  |  |  |  |  |
|  | *Sa*10^4^ (n=21)r1 | 8 | 11 | 2 | 0 | 0 | 21 | 0 | .046 | .326 | .020 | 1 | *.889* | 1.047 |
|  | *Sa*10^4^ (n=17)r2 | 6 | 9 | 2 | 0 | 0 | 17 | 0 |  |  |  |  |  |  |
|  | *Pl*10^5^ (n=14)r1 | 0 | 3 | 6 | 3 | 2 | 14 | 0 | -.366 | .395 | .857 | 1 | *.355* | .694 |
|  | *Pl*10^5^ (n=14)r2 | 0 | 5 | 6 | 3 | 0 | 14 | 0 |  |  |  |  |  |  |
|  | | | | | | | | | | | | | | |
| **Pl** | *Ec*10^6^ (n=16)r1 | 12 | 0 | 0 | 3 | 0 | 15 | 1 | -.195 | .413 | .224 | 1 | *.636* | .822 |
|  | *Ec*10^6^ (n=10)r2 | 9 | 0 | 0 | 1 | 0 | 10 | 0 |  |  |  |  |  |  |
|  | *Sa*10^4^ (n=25)r1 | 10 | 14 | 1 | 0 | 0 | 25 | 0 | -.005 | .295 | .000 | 1 | *.987* | .995 |
|  | *Sa*10^4^ (n=22)r2 | 8 | 14 | 0 | 0 | 0 | 22 | 0 |  |  |  |  |  |  |
|  | *Pl*10^5^ (n=35)r1 | 0 | 1 | 12 | 22 | 0 | 35 | 0 |  |  | .169 | 3 | *.982* |  |
|  | *Pl10^5^ (n=30)r2* | 0 | 0 | 13 | 17 | 0 | 30 | 0 |  |  |  |  |  |  |
|  | *Pl10^5^ (n=31)r3* | 0 | 0 | 15 | 16 | 0 | 31 | 0 |  |  |  |  |  |  |
|  | *Pl10^5^ (n=33)r4* | 0 | 0 | 14 | 19 | 0 | 33 | 0 |  |  |  |  |  |  |

**Table S3**. Cumulative mortality at the end of the first week after the challenge and before the infection phase started.

| **Challenge type** | **Nr. of bees per group** | **Nr. of dead bees at the end of the week** | **Cumulative mortality (%)** |
| --- | --- | --- | --- |
| Naive | 100 | 4 | 4 |
| Ringer | 343 | 18 | 5,25 |
| Heat-killed *Pl* | 273 | 11 | 4,03 |
| Heat-killed *Ec* | 137 | 7 | 5,11 |
| Heat-killed *Sa* | 120 | 4 | 3,33 |

**Table S4.** Means and medians for survival time of honeybee groups expose to different kinds of challenge type and infection.

|  | | | | | | | | | |
| --- | --- | --- | --- | --- | --- | --- | --- | --- | --- |
| challenge type | infection | Mean^a^ | | | | Median | | | |
|  |  | Estimate | Std. Error | 95% Confidence Interval | | Estimate | Std. Error | 95% Confidence Interval | |
|  |  |  |  | Lower Bound | Upper Bound |  |  | Lower Bound | Upper Bound |
| Ringer | Ringer | 4.928 | .044 | 4.841 | 5.015 | . | . | . | . |
|  | ***Pl*** | **2.736** | .042 | 2.654 | 2.819 | **3.000** | .015 | 2.970 | 3.030 |
|  | ***Ec*** | 1.471 | .135 | 1.205 | 1.736 | 1.000 | . | . | . |
|  | ***Sa*** | 1.660 | .082 | 1.499 | 1.820 | 2.000 | .051 | 1.900 | 2.100 |
|  | Overall | 2.952 | .081 | 2.794 | 3.110 | 3.000 | .083 | 2.838 | 3.162 |
| heat-killed *Pl* | Ringer | 4.887 | .058 | 4.773 | 5.001 | . | . | . | . |
|  | ***Pl*** | **3.566** | .045 | 3.477 | 3.654 | **4.000** | .000 | . | . |
|  | ***Ec*** | 1.615 | .250 | 1.126 | 2.105 | 1.000 | . | . | . |
|  | ***Sa*** | 1.638 | .077 | 1.487 | 1.789 | 2.000 | .035 | 1.931 | 2.069 |
|  | Overall | 3.392 | .082 | 3.232 | 3.552 | 4.000 | .105 | 3.794 | 4.206 |
| heat-killed *Ec* | Ringer | 4.829 | .103 | 4.628 | 5.031 | . | . | . | . |
|  | ***Pl*** | 3.071 | .170 | 2.738 | 3.405 | 3.000 | .199 | 2.610 | 3.390 |
|  | ***Ec*** | 1.935 | .207 | 1.530 | 2.341 | 1.000 | . | . | . |
|  | ***Sa*** | 1.737 | .105 | 1.532 | 1.942 | 2.000 | .095 | 1.815 | 2.185 |
|  | Overall | 2.971 | .132 | 2.713 | 3.229 | 3.000 | .203 | 2.602 | 3.398 |
| heat-killed *Sa* | Ringer | 4.844 | .126 | 4.597 | 5.090 | . | . | . | . |
|  | ***Pl*** | 3.148 | .231 | 2.696 | 3.601 | 3.000 | .350 | 2.314 | 3.686 |
|  | ***Ec*** | 2.382 | .203 | 1.985 | 2.779 | 2.000 | .287 | 1.438 | 2.562 |
|  | ***Sa*** | 1.974 | .133 | 1.712 | 2.235 | 2.000 | .132 | 1.741 | 2.259 |
|  | Overall | 3.023 | .130 | 2.768 | 3.278 | 3.000 | .192 | 2.623 | 3.377 |
| Overall | Overall | 3.107 | .049 | 3.010 | 3.204 | 3.000 | .075 | 2.853 | 3.147 |
| a. Estimation is limited to the largest survival time if it is censored. | | | | | | | | | |

**Table S5.** Bonferroni pos-hoc multiple comparisons for size of the zone of inhibition using hemolymph of challenged honeybees.

| (I) group | (J) group | Mean Difference (I-J) | Std. Error | Sig. | 95% Confidence Interval | |
| --- | --- | --- | --- | --- | --- | --- |
|  |  |  |  |  | Lower Bound | Upper Bound |
| naive | Ringer | -2.6333^*^ | .46680 | .000 | -3.8811 | -1.3856 |
|  | *Pl* | -5.2083^*^ | .41633 | .000 | -6.3212 | -4.0955 |
| Ringer | naive | 2.6333^*^ | .46680 | .000 | 1.3856 | 3.8811 |
|  | *Pl* | -2.5750^*^ | .43947 | .000 | -3.7497 | -1.4003 |
| *Pl* | naive | 5.2083^*^ | .41633 | .000 | 4.0955 | 6.3212 |
|  | Ringer | 2.5750^*^ | .43947 | .000 | 1.4003 | 3.7497 |
| Based on observed means.  The error term is Mean Square(Error) = ,594. | | | | | | |
| *. The mean difference is significant at the 0,05 level. | | | | | | |

**Table S6.** Mean weight by insect and treatment. Note that weight is given in grams. Data are shown as observed means and SDs.

|  | Treatment | | | | | |  | |  |
| --- | --- | --- | --- | --- | --- | --- | --- | --- | --- |
|  | Naive | | Ringer | | PL | | Total | | |
|  | M | SD n | M | SD n | M | SD n | M | SD n | |
| Bumblebees | 0.25 | 0.07 10 | 0.22 | 0.04 9 | 0.23 | 0.04 12 | 0.23 | 0.05 31 | |
| Honeybees | 0.12 | 0.01 15 | 0.13 | 0.01 13 | 0.12 | 0.01 14 | 0.12 | 0.01 42 | |
| Total | 0.17 | 0.08 | 0.16 | 0.05 | 0.17 | 0.07 | 0.17 | 0.07 | |

**Table S7**. Summary table of flight performance of honeybees and bumblebees. Mean and SD are given for all variables for the three experimental groups in bumblebees and honeybees. Note that data are adjusted for the covariate weight. 95% confidence level around the mean is given in parenthesis.

|  |  | Treatment | | |  |
| --- | --- | --- | --- | --- | --- |
|  |  | Naive  M ± SD  (95% CI) | Ringer  M ± SD  (95% CI) | *Pl*  M ± SD  (95% CI) | Total  M ± SD  (95% CI) |
| Bumblebees | Flight duration (min) | 36.62 ± 11.10  (29.61, 43.63) | 26.74 ± 9.02  (20.74, 32.75) | 22.28 ± 10.25  (16.37, 28.19) | 28.55 ± 13.15  (23.83, 33.26) |
|  | Covered distance (m) | 2714.11 ± 995.96  (2085.29, 3342.93) | 1959.93 ± 809.24  (1421.36, 2498.49) | 1657.95 ± 919.64  (1127.90, 2187.99) | 2110.66 ± 1179.51  (1687.70, 2533.63) |
|  | Maximum speed (m/s) | 1.85 ± 0.57  (1.49, 2.21) | 1.55 ± 0.46  (1.24, 1.86) | 1.63 ± 0.52  (1.33, 1.94) | 1.68 ± 0.67  (1.44, 1.92) |
|  | Average speed (m/s) | 1.30 ± 0.44  (1.02, 1.58) | 1.10 ± 0.36  (0.86, 1.34) | 1.18 ± 0.41  (0.95, 1.42) | 1.19 ± 0.52  (1.01, 1.38) |
| Honeybees | Flight duration (min) | 25.91 ± 9.78  (20.87, 30.95) | 24.35 ± 9.13  (19.29, 29.41) | 25.97 ± 9.89  (20.69, 31.24) | 25.41 ± 12.03  (21.70, 29.12) |
|  | Covered distance (m) | 1877.95 ± 877.02  (1425.83, 2330.06) | 1787.35 ± 818.93  (1333.87, 2240.83) | 1946.98 ± 887.20  (1473.56, 2420.39) | 1870.76 ± 1079.54  (1538.18, 2203.34) |
|  | Maximum speed (m/s) | 1.54 ± 0.50  (1.28, 1.80) | 1.46 ± 0.47  (1.20, 1.72) | 1.51 ± 0.51  (1.24, 1.78) | 1.50 ± 0.62  (1.31, 1.69) |
|  | Average speed (m/s) | 1.21 ± 0.39  (1.01, 1.41) | 1.15 ± 0.36  (0.95, 1.35) | 1.23 ± 0.39  (1.02, 1.44) | 1.20 ± 0.48  (1.05, 1.34) |
| Total | Flight duration (min) | 31.27 ± 8.61  (27.83, 34.71) | 25.55 ± 8.23  (22.05, 29.05) | 24.12 ± 8.14  (20.93, 27.31) |  |
|  | Covered distance (m) | 2296.03 ± 772.3  (1987.64, 2604.42) | 1873.64 ± 738.01  (1559.49, 2187.79) | 1802.46 ± 730.29  (1516.51, 2088.41) |  |
|  | Maximum speed (m/s) | 1.69 ± 0.44  (1.52, 1.87) | 1.50 ± 0.42  (1.32, 1.68) | 1.57 ± 0.42  (1.41, 1.74) |  |
|  | Average speed (m/s) | 1.25 ± 0.35  (1.12, 1.39) | 1.13 ± 0.33  (0.99, 1.27) | 1.20 ± 0.33  (1.08, 1.33) |  |
